# Nursing community in Croatia and academic writing: a continuous challenge

**DOI:** 10.3325/cmj.2013.54.504

**Published:** 2013-10

**Authors:** Višnja Vičić-Hudorović, Narcis Hudorović

**Affiliations:** 1 Editor in Chief of Sestrinski glasnik/Nursing Journal, Croatian Nurses Association, Zagreb, Croatia; 2 Department of Vascular Surgery, University Clinical Center “Sestre milosrdnice,” Zagreb, Croatia

To the Editor,

We read the article by Kalauz et al (1) with great interest. As authors stated, in Croatian nursing community there is a need for scientific journal of nursing which would enable nurses to publish scientific publications necessary to disseminate knowledge and obtain references for professional and scientific advancement within the profession. In new disciplines, such as nursing science, writing is an effective tool in constructing the culture of a new research community.

Nursing science has been gradually built as a result of a great deal of research, thought, and principled analysis bringing together nursing and other disciplines. The body of nursing science is formed by best practices, ambitious vision, and high ideals. All of this propels nursing toward being a professional rather than a vocational skill (2).

Vocational nursing is criticized for being rote, lacking vision, and scientific approach. On the other hand, university nursing programs teach grad nurses so much information that they cannot see the distinction between vocational and scientific work (3).

Today, nursing science is a body of knowledge that the nursing communities and their professional associations have deemed important and desirable, and serves the future development of the profession. An even more important issue, though, is the possibility of nursing science to influence nursing practice. Clinical nurses must find the easiest way to access nursing research and know how to apply the findings in their everyday practice (4). Moreover, clinical nurses have rarely the time to seek and read all different research reports about the topics of relevance for them, and they find reading and critiquing research reports difficult. Even if they try, they will frequently find that different research reports about the same topic will lead to different conclusions. They are then faced with the question of how to overcome the inconsistencies in the literature. The danger of this situation is that nursing research is not considered by clinical nurses a resource of relevant knowledge that helps them better understand the phenomena observed in the practice or to solve problems they encounter. In order to overcome such a situation, it is the task of nursing scientific journals to present comprehensive and critically evaluated articles. To fulfill all these demands, Journal Sestrinski glasnik/Nursing Journal, recently introduced some changes (new Web site, a new cover design, changes in the layout, article type), which could contribute to better presentation of nursing articles, which could be useful both to the nurses and the nursing university community in Croatia (5).
